# Modeling the Combined Effect of Pressure and Mild Heat on the Inactivation Kinetics of *Escherichia coli, Listeria innocua*, and *Staphylococcus aureus* in Black Tiger Shrimp (*Penaeus monodon*)

**DOI:** 10.3389/fmicb.2017.01311

**Published:** 2017-07-24

**Authors:** Barjinder P. Kaur, P. Srinivasa Rao

**Affiliations:** ^1^Department of Food Engineering, National Institute of Food Technology Entrepreneurship and Management Sonepat, India; ^2^Agricultural and Food Engineering Department, Indian Institute of Technology Kharagpur Kharagpur, India

**Keywords:** black tiger shrimp, high-pressure processing, inactivation, pathogens, weibull, log-linear

## Abstract

The high-pressure inactivation of *Escherichia coli, Listeria innocua*, and *Staphylococcus aureus* was studied in black tiger shrimp (*Penaeus monodon*). The processing parameters examined included pressure (300 to 600 MPa) and temperature (30 to 50°C). In addition, the pressure-hold period (0 to 15 min) was investigated, thus allowing both single-pulse pressure effects (i.e., zero holding time) and pressure-hold effects to be explored. *E. coli* was found to be the most sensitive strain to single-pulse pressure, followed by *L. innocua* and lastly *S. aureus*. Higher pressures and temperatures resulted in higher destruction rates, and the value of the shape parameter (β′) accounted for the downward concavity (β′ > 1) of the survival curves. A simplified Weibull model described the non-linearity of the survival curves for the changes in the pressure-hold period well, and it was comparable to the original Weibull model. The regression coefficients (*R*^2^), root mean square error (RMSE), accuracy factor (*A*_*f*_), bias factor (*B*_*f*_), and residual plots suggested that using linear models to represent the data was not as appropriate as using non-linear models. However, linear models produced good fits for some pressure–temperature combinations. Analogous to their use in thermal death kinetics, activation volume (*V*_*a*_) and activation energy (*E*_*a*_) can be used to describe the pressure and temperature dependencies of the scale parameter (δ, min), respectively. The *V*_*a*_ and *E*_*a*_ values showed that high pressure and temperaturefavored the inactivation process, and *S. aureus* was the most baro-resistant pathogen.

## Introduction

High-pressure processing (HPP) offers an attractive alternative to heat pasteurization as a means to produce preservative-free, microbiologically safe, and stable foods (Pavuluri and Kaur, 2014). Over the last decade, significant progress has been made in the high-pressure pasteurization of foods, and several commercial products treated with HPP are now available on the market in several countries (Balasubramaniam et al., 2008). The high level of interest in this novel technology is due to its ability to destroy or inactivate foodborne microorganisms and enzymes in food with minimal change in the organoleptic properties and nutritional quality of the food. Pressures ranging from 300 to 600 MPa can inactivate most pathogenic and spoilage vegetative cells, yeasts, and molds (Smelt, 1998). Moreover, it is economically beneficial to use lower pressure in combination with mild heat (30–50°C) (Buzrul et al., 2008).

For effective use of high-pressure technology for food preservation, it is necessary to study the interactions between the processing parameters (pressure, time, and temperature) and to determine the optimum conditions for obtaining desirable levels of microbial destruction while maintaining a high degree of nutritional quality and good flavor and texture (Kaur and Rao, 2016).

Several studies have demonstrated the efficacy of HPP for inactivating a wide spectrum of Gram-negative and Gram-positive bacteria in suspensions, as well as in solid food items. HPP inactivates microorganisms by acting against multiple targets, including intracellular and membrane-bound enzymes (Wouters et al., 1998). However, the rate and pattern of HPP-induced microorganism inactivation is quite variable and influenced by the processing conditions, medium composition, and microorganism type/strain. Therefore, there is a need for accurate prediction of the inactivation behavior of foodborne microorganisms, as well as accurate characterization of their resistance to HPP. Kinetic modeling is helpful for determining the most efficient processing parameters and for the prediction of the HPP effects on microbial inactivation and product shelf-life. It is also an effective way of carrying out a risk assessment and simulation of the HPP process (Smelt et al., 2002).

Although simple first-order-type inactivation curves do sometimes occur for pressure-treated cells (Basak et al., 2002; Riahi et al., 2003; Dogan and Erkmen, 2004; Ramaswamy et al., 2008), significant deviations from linearity (such as, sigmoidal curves, curves with shoulders, and tailing) have been reported by multiple researchers (Garriga et al., 2002; Chen and Hoover, 2003; Buzrul and Alpas, 2004; Rajan et al., 2006; Ahn et al., 2007; Rendueles et al., 2011). A number of models have been proposed to describe these non-linear survival curves, such as, the Baranyi, Weibull, modified Gompertz, log-logistic, and quasi-chemical models. Among these models, the most simple and flexible is the Weibull model, and it is reported to fit the experimental data better than the other models (Panagou et al., 2007; Serment-Moreno et al., 2014).

Black tiger shrimp (*Penaeus monodon*) is a commercially important shrimp species, persistently in demand in the global market due to its distinct flavor, texture, and high nutritional value (Pushparajan and Soundarapandian, 2010). In past decades, there have been many issues associated with shrimp exported from India due to high bacteria counts and the presence of pathogenic bacteria such as, *E. coli* and *Listeria monocytogenes. L. monocytogenes* is particularly problematic for the food industry as it is widespread in the environment and can grow in a broad range of temperatures (Jofré et al., 2009). Crustaceans have also been linked with food poisoning attributed to *S. aureus* because of the ubiquity of this organism in the processing environment combined with the degree of handling required for processing these food types (Nicolaides, 2009).

Thus, the present study was undertaken with the aim of investigating the inactivation kinetics of *E. coli* O157:H7 ATCC 43895 (a Gram-negative bacterium) and two Gram-positive bacteria, namely *L. innocua* ATCC 33090 (a nonpathogenic indicator microorganism for *L. monocytogenes*) and *S. aureus* ATCC 29213, in black tiger shrimp at various pressure–temperature (*P–T*) combinations. These three foodborne pathogens have been reported to be resistant to pressure treatment (Guan et al., 2006; Buzrul et al., 2008; Ruiz-Espinosa et al., 2013).

## Materials and methods

### Preparation of bacterial cultures

Pure cultures *of E. coli* O157:H7 ATCC 43895, *L. innocua* ATCC 33090, and *S. aureus* ATCC 29213 were procured in freeze-dried form from Merck Ltd India. The bacteria were activated by inoculating tryptic soy broth supplemented with 0.6% yeast extract (TSBYE) (product number: M011; Himedia Laboratories, India) with each of the pure cultures, and they were then incubated at 37°C for 24 h. Stock cultures were prepared by mixing the activated cultures with sterilized 80% glycerol (v/v) in a 1:1 ratio in Eppendorf tubes, which were stored at −40°C in a deep freezer until further analysis. The purity of the cultures was determined by Gram staining and microscopic observation. Each inoculum was prepared by inoculating 100 mL of sterile TSBYE with 1 mL of the thawed stock culture, and it was then incubated at 37°C for 24–30 h to obtain a count of 10^9^ colony-forming units (CFU) mL^−1^.

### Preparation and inoculation of samples

Freshly harvested black tiger shrimp weighing 25–35 g were obtained from Shankarpur coast, West Bengal, India, and transported to the laboratory within 4 h under chilled conditions (with a sample-to-ice ratio of 1:1). The shrimp were washed with chilled water, deheaded, and shelled. Before inoculation, the samples were tested for the presence of *E. coli, Listeria*, and *S. aureus* according to the method proposed by the APHA (2001), which is described later in Section Enumeration.

After ensuring the absence of these organisms, test samples were inoculated with the activated bacteria, according to the method described by Anang et al. (2007). Whole shrimp samples were dipped into each of the prepared inocula (10^9^ CFU mL^−1^) individually at room temperature (27°C) for 15 min. The ratio of sample to culture suspension was 1:2 (w/v), which allowed complete immersion of the samples. The initial bacterial cell counts in the shrimp samples after dipping were approximately 10^7^ CFU g^−1^. Thereafter, the samples were left to dry in a sterile cabinet for 1 h. The inoculated samples were packed in ethylene vinyl alcohol films (thickness: 110 ± 1.0 μm), with three shrimps per pouch. The samples were double packed to ensure no direct contact with the pressure-transmitting medium during the HPP.

### HPP

The high-pressure treatment was performed in a lab-scale HPP unit (model: S-IL-100-250-09-W; Stansted Fluid Power Systems, UK), with aqueous monopropylene glycol 30% (v/v) as the pressure-transmitting medium. The samples were processed at four pressures, 300, 400, 500, and 600 MPa, and four temperatures, 30, 40, 50, and 60°C. For each *P–T* combination, the following six pressure-hold periods were investigated: 0, 3, 6, 9, 12, and 15 min. The single-pulse pressurization (0-min holding time) involved pressurization of the sample to the desired level followed by immediate depressurization. The rate of pressurization was fixed at 300 MPa min^−1^ and the depressurization was achieved in <10 s. After *P–T* treatment, the pouches were kept at 4°C for 24 h to allow the pressurized cells to recover from the pressure stress effect before analysis. For each *P–T* combination, three pouches (with three shrimp per pouch) were processed. The experiments were performed independently twice and, for each processing condition, three samples were analyzed.

### Enumeration

Microbiological analyses were performed according to the method described by the APHA (2001). A 10-g sample was aseptically cut from each sample lot and macerated with 90 mL peptone water (0.1%) in a sterile glass mortar. The homogenate was serially diluted using 0.1% sterile peptone water and plated onto appropriate culture media. For ease of handling, the direct pour plate method was adopted. *E. coli* was enumerated on Violet Red Bile Agar and *L. innocua* on Listeria-Selective Agar Base (PALCAM). The plates were incubated at 37°C for 24 h for *E. coli* and 48 h for *L. innocua*. To examine the surviving *S. aureus* populations, samples were inoculated on Baird Parker Agar and the plates incubated at 37°C for 48 h. Black colonies with clear zones, which were presumed to be *S. aureus*, were counted.

### Kinetics analysis

The pressure-induced destruction of the microorganisms was analyzed in terms of the single-pulse pressure effect (SPPE) and the pressure-hold effect. First, the SPPE was determined by calculating the logarithmic difference between the initial microbial counts in the control samples (*N*_0_) and the number of cells that survived after single-pulse pressurization (*N*_*PE*_) as follows:

(1) $$\begin{array}{r} SPPE=Log_{10}\left(N_{0}\right)-Log_{10}\left(N_{PE}\right) \end{array}$$

Second, a modified Weibull model (Equation 2; (Chakraborty et al., 2015)) was fitted to the data on survivor fractions corresponding to the different pressure-hold periods using Origin Pro version 8.0 (OriginLab Corporation, Northampton, MA, USA).

(2) $$\begin{array}{r} Log_{10}\frac{N_{t}}{N_{PE}}=-{\left(\frac{t}{\delta^{\prime }}\right)}^{\beta^{\prime }} \end{array}$$

where *N*_*t*_ is the number of microorganisms that survived after pressure treatment for time *t* (min), *N*_*PE*_ is the number of cells that survived after single-pulse pressurization, and δ′ and β′ represent the scale parameter (min) and shape parameter (dimensionless), respectively. β′ < 1 denotes upward concavity, β′ > 1 represents downward concavity, and β′ = 1 corresponds to linear (first-order) kinetics. The concavity can be used to interpret the inactivation resistance of the bacterial population: (a) homogenous (β′ = 1), (b) increasing resistance (β′ < 1), or (c) decreasing resistance as a result of accumulated damage in the population (β′ > 1) (Serment-Moreno et al., 2014).

The best-fit values of δ′ and β′ were determined for each species at each of the *P–T* combinations. It has been reported that the shape parameter (β′) represents the microorganism behavior index (Fernandez et al., 2002). Therefore, within the ranges of the processing parameters evaluated in this study, for each species, β was fixed at a uniform value using the method described by Chakraborty et al. (2015). In brief, for a pre-assumed value of β′, the δ′ values were calculated using Equation (3) for the five non-zero pressure-hold periods (with three replications for each *P–T* combination).

(3) $$\begin{array}{r} \delta^{\prime }=\left[\frac{t}{\left\{-Log_{10}{\left(\frac{N_{t}}{N_{PE}}\right)}^{\frac{1}{\beta^{\prime }}}\right\}}\right] \end{array}$$

Next, the logarithm of the fractional count value (–Log_10_N_t_/N_PE_) was recalculated using Equation (2) at the average value of δ′ (δ′_avg_, min) for the relevant *P–T* combination. For each species, the value of β′ was varied from 0 to 2 and the value with the minimum cumulative sum of the square of errors (SSE; according to Eq. 4) was designated as the uniform β for the entire *P–T* domain tested.

(4) $$SE=\sum \;{\left[{\left(Log_{10}\frac{N_{t}}{N_{PE}}\right)}_{\delta_{avg}^{\prime }}-{\left(Log_{10}\frac{N_{t}}{N_{PE}}\right)}_{experimental}\right]}^{2}$$

After fixing the β value at a single value for the entire *P–T* domain, the δ values were recalculated for all the conditions tested. Analogous to thermal death kinetics, using δ to represent the decimal reduction time (*D*-value, min), the rate of destruction (*k*, min^−1^) was calculated according to Equation (5).

(5) $$\begin{array}{r} k={\left(\frac{2.303}{\delta^{\beta }}\right)}^{\frac{1}{\beta }} \end{array}$$

The pressure sensitivity of *k* at a fixed temperature was quantified using the activation volume (*V*_*a*_, cm^3^mol^−1^), which was calculated using the Eyring equation (Equation 6).

(6) $$\begin{array}{r} \text{Ln }k=\text{Ln }k_{ref,P}+\frac{V_{a}}{RT}\left[P_{ref}-P\right] \end{array}$$

where *k*_*ref, P*_ is the rate constant (min^−1^) at the reference pressure, *P*_*ref*_ (450 MPa, the midpoint of the pressure axis).

The temperature sensitivity of *k* at a fixed pressure was quantified using the activation energy (*E*_*a*_, kJ mol^−1^). This was calculated using the Arrhenius equation (Equation 7) in which the logarithmic function of *k* is directly proportional to the reciprocal of the absolute temperature (*T*, K).

(7) $$\begin{array}{r} \text{Ln }k=\text{Ln }k_{ref,T}+\frac{E_{a}}{R}\left[\frac{1}{T_{ref}}-\frac{1}{T}\right] \end{array}$$

where *k*_*ref, T*_ is the rate constant (min^−1^) at the reference temperature, *T*_*ref*_ (318 K, the midpoint of the temperature axis).

The regression analyses for calculating the rate constants, *V*_a_, *E*_*a*_, SSE, and regression coefficients (*R*^2^) were performed in Microsoft Excel 2007 (Microsoft Corp., USA). Non-linear curve fitting for *k* as a function of *V*_*a*_ or *E*_a_ was performed using Origin Pro version 8.0.

### Statistical analysis

An analysis of variance (ANOVA) was carried out using SPSS for Windows version 17 (SPSS Inc., Chicago, IL, USA). The difference between the pairs of means was evaluated using Tukey's test, with a *P*–value < 0.05 being considered statistically significant.

## Results and discussion

### Effect of single-pulse pressurization

A significant SPPE (*P* < 0.05) was observed, both with increases in pressure and temperature, indicating the efficacy of single-pulse pressurization for lowering microbial counts (Table 1). For processing parameters of 300–600 MPa/30–60°C, the SPPE values ranged from 0.90 to 4.29 Log_10_ cycles, 0.88 to 4.39 Log_10_ cycles, and 0.75 to 5.61 Log_10_ cycles for *E. coli, L. innocua*, and *S. aureus*, respectively. The effect of temperature was observed to be statistically more significant (*P* < 0.05) than that of pressure. Among the three microorganisms studied, *E. coli* exhibited the greatest sensitivity to single-pulse pressure, followed by *L. innocua* and lastly *S. aureus*. Ramaswamy et al. (2008) reported similar findings, with greater inactivation by pulse pressurization of *E. coli* O157:H7 compared to *L. monocytogenes* Scott A.

**Table 1:** Estimated PE values (Log PE) for *E. coli, L. innocua* and *S. aureus* in black tiger shrimp.

**Bacterial species**	**Temperature(°C)**	**Pressure (MPa)**			
		**300**	**400**	**500**	**600**
*E. coli*	30	0.90 ± 0.04^aA^	1.15 ± 0.03^aA^	1.56 ± 0.10^aA^	1.75 ± 0.09^aA^
	40	2.74 ± 0.09^aB^	3.17 ± 0.11^abB^	3.99 ± 0.08^bB^	4.29 ± 0.17^bB^
*L. innocua*	30	0.88 ± 0.04^aA^	1.09 ± 0.06^aA^	1.46 ± 0.14^aA^	1.58 ± 0.08^aA^
	40	2.65 ± 0.15^aB^	2.99 ± 0.14^aB^	4.01 ± 0.23^bB^	4.39 ± 0.11^bB^
*S. aureus*	30	0.75 ± 0.05^aA^	0.90 ± 0.07^aA^	1.23 ± 0.05^aA^	1.29 ± 0.08^aA^
	40	2.09 ± 0.14^aB^	2.69 ± 0.2^abB^	3.73 ± 0.09^bB^	4.01 ± 0.17^bB^
	50	4.39 ± 0.11^aC^	4.81 ± 0.21^abC^	5.19 ± 0.25^bC^	5.61 ± 0.20^bC^

*All values are means ± standard deviations of data from two independent experiments. Different superscripts (a, b) in the same row indicate significant difference (P < 0.05). Different superscripts (A, B, C) in the same column indicate significant difference (P < 0.05)*.

The reductions in the bacterial counts induced by single-pulse pressurization were due to the formation of cavitation voids in the bacterial cells as a result of rapid pressurization and depressurization, which led to physical disruption and death of the bacteria (Hiremath and Ramaswamy, 2011). The amount of disruption to the cell wall is dependent on the processing conditions (Ramaswamy et al., 2003). The depressurization magnitude has been reported to have a greater effect on the cell wall than the pressurization magnitude (Hayakawa et al., 1994). The increase in pressure come up time (i.e., the time required to reach the desired pressure) caused the microorganisms to be exposed to pressurization stress for an extra period of time, which increased the extent of inactivation. In addition, the greater reduction in bacterial counts at higher temperature was due to thermal lethality, which thus aided the damage caused by the pressure treatment (Chakraborty et al., 2014).

### Isobaric–isothermal inactivation kinetics of pathogens

**Figure 1:**
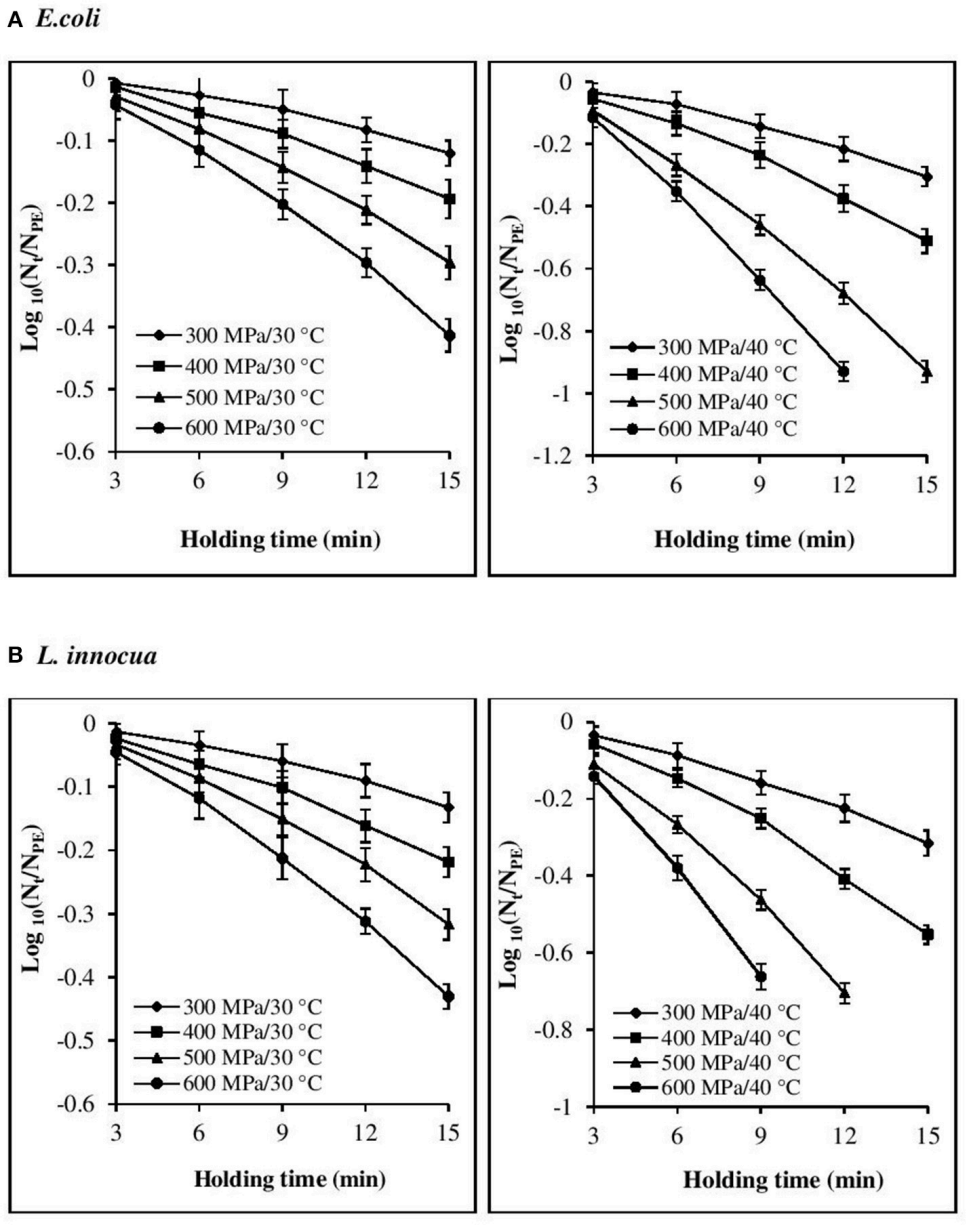
Effect of different pressure-temperature treatments on the inactivation of **(A)** *E. coli* and **(B)** *L. innocua* in black tiger shrimp.

**Figure 2:**
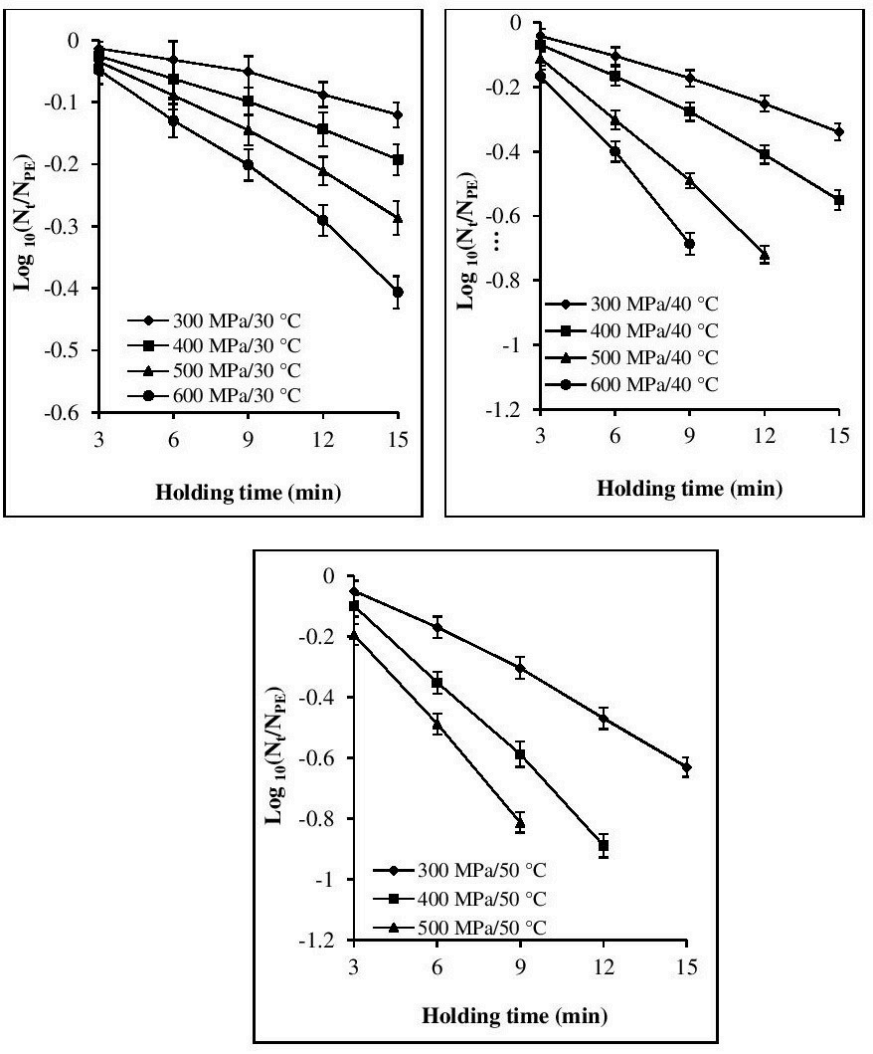
Effect of different pressure-temperature treatments on the inactivation of *S. aureus* in black tiger shrimp.

The inactivation rate increased with increases in each of the three processing parameters, viz. pressure, temperature, and time (Figures 1, 2). The inactivation rates of the three pathogens during isobaric–isothermal cycles decreased in the following order: *L. innocua* > *E. coli* > *S. aureus*. Several researchers have also reported that *S. aureus* was the most baro-resistant pathogen in their studies (Yuste et al., 2004; Jofré et al., 2009; Cebrián et al., 2016).

At 30°C, the reduction in the count of all three pathogens was <5 Log CFU g^−1^. The maximum inactivation, 5 Log CFU g^−1^ was obtained for *L. innocua* at 600 MPa/15 min. *S. aureus* exhibited the highest resistance to pressure, as a minimum processing intensity of 500 MPa/9 min/50°C was required for its complete destruction. However, in the case of *L. innocua*, no detectable levels were observed after treatment at 500 MPa/15 min/40°C and 600 MPa/12–15 min/40°C. In contrast, *E. coli* was completely destroyed at 600 MPa/15 min/40°C. At 50°C, neither organism was detected for any of the pressure-holding time conditions. Enhancement of pathogen viability loss with increases in pressure and temperature has been reported for multiple products, including broth (Alpas et al., 2000), milk (Gao and Jiang, 2005), juices (Van Opstal et al., 2003; Bayindirli et al., 2006), and ham (Tassou et al., 2008). The inactivation rates achieved in these studies varied due to the differences in treatment conditions, bacterial strain, test substrate, and enumeration medium.

### Model fitting for the isobaric period

Visual inspection of the isothermal–isobaric inactivation kinetic curves of the studied pathogens suggested that the curves were non-linear (β ≠ 1) in most cases (Figures 1, 2). Therefore, fitting a straight line to the data points was deemed inappropriate as it would result in considerable errors. Non-linearity in the survival curve might arise from the adaptability of bacterial subpopulations, with variable resistance to the applied stress, which also depends on the surrounding conditions (Bevilacqua et al., 2015).

At all the *P–T* combinations, the logarithmic survival curves showed the same trend, with downward concavity (β′ > 1). The downward concavity suggested that the microbial cells became less resistant with increased pressure-hold periods, which might be due to the separation of the bacterial cells into clumps (Chakraborty et al., 2015). Based on the minimum SSE values obtained, the β values were set at 1.48, 1.4, and 1.3 for *E. coli, L. innocua*, and *S. aureus*, respectively, for the entire *P–T* domain used in the study. The β values were significantly different (*p* < 0.05) from β_*l*_; however, there was no significant difference in comparison to β′ (Table 2). Using fixed β values resulted in simpler models, and the reduction in the number of parameters is expected to have resulted in more reliable predictions.

**Table 2:** Shape factor (β_*l*_, β′, and β) obtained after model fitting at different pressure-temperature combinations for different microorganisms in black tiger shrimp.

**Bacterial species**	**Pressure (MPa)**	**The log-linear model**		**The Weibull models**							
		**β_*l*_ = 1**		**30°C**		**40°C**		**50°C**		**Overall β at SSE_min_**	
		**β_*l*_**	**Adj *R*^2^ at 30, 40, 50°C**	**β′**	**Adj *R*^2^**	**β′**	**Adj R*^2^***	**β′**	**Adj *R*^2^**	**β**	**Adj *R*^2^ at 30, 40, 50°C**
*E. coli*	300	1^a^	0.90, 0.91	1.45 ± 0.05^b^	0.99	1.49 ± 0.07^b^	0.97	ND	ND	1.48^b^	0.99, 0.98
	400	1^a^	0.89, 0.86	1.44 ± 0.07^b^	0.98	1.46 ± 0.02^b^	0.94	ND	ND	1.48^b^	0.97, 0.97
	500	1^a^	0.78, 0.80	1.51 ± 0.11^b^	0.99	1.42 ± 0.02^ab^	0.95	ND	ND	1.48^b^	0.96, 0.97
	600	1^a^	0.76, 0.81	1.52 ± 0.02^b^	0.99	1.43 ± 0.05^a^	0.97	ND	ND	1.48^b^	0.98, 0.97
*L. innocua*	300	1^a^	0.87, 0.87	1.41 ± 0.09^b^	0.99	1.39 ± 0.05^b^	0.98	ND	ND	1.40^b^	0.96, 0.98
	400	1^a^	0.88, 0.86	1.39 ± 0.05^b^	0.98	1.41 ± 0.05^b^	0.97	ND	ND	1.40^b^	0.98, 0.98
	500	1^a^	0.85, 0.83	1.43 ± 0.04^b^	0.98	1.39 ± 0.11^b^	0.99	ND	ND	1.40^b^	0.98, 0.99
	600	1^a^	0.85, 0.85	1.40 ± 0.04^b^	0.97	1.39 ± 0.06^b^	0.97	ND	ND	1.40^b^	0.97, 0.98
*S. aureus*	300	1^a^	0.85, 0.89, 0.86	1.33 ± 0.08^b^	0.99	1.31 ± 0.07^b^	0.96	1.34 ± 0.07^b^	0.98	1.30^b^	0.97, 0.98, 0.97
	400	1^a^	0.82, 0.80, 0.82	1.29 ± 0.10^b^	0.98	1.32 ± 0.03^b^	0.98	1.28 ± 0.07^b^	0.97	1.30^b^	0.98, 0.99, 0.99
	500	1^a^	0.87, 0.81, 0.81	1.32 ± 0.02^b^	0.99	1.30 ± 0.06^b^	0.97	1.30 ± 0.07^b^	0.98	1.30^b^	0.98, 0.98, 0.99
	600	1^a^	0.86, 0.78, 0.81	1.35 ± 0.03^b^	0.99	1.31 ± 0.05^b^	0.98	ND	ND	1.30^b^	0.98, 0.98, 0.99

*All values are means ± standard deviations of data from two independent experiments. Different superscripts (a, b) in the same row indicate significant difference (P < 0.05). ND, not determined*.

The comparison of the model parameters obtained for the log-linear and both Weibull models at different temperatures is presented in Tables 2, 3. The simplified Weibull model produced fits comparable to the original Weibull model. The goodness of fit of the models was compared by computing the adjusted *R*^2^ and RMSE, along with the accuracy factor (*A*_*f*_) and bias factor (*B*_*f*_) (Ross, 1996), which are shown in Equations (8) and (9), respectively, in which *N* represents the number of *k*-values estimated.

(8) $$\begin{array}{r} A_{f}=10^{\frac{\sum |Log\left(\frac{k_{predicted}}{k_{observed}}\right)|}{N}} \end{array}$$

(9) $$\begin{array}{r} B_{f}=10^{\frac{\sum Log\left(\frac{k_{predicted}}{k_{observed}}\right)}{N}} \end{array}$$

**Table 3:** Comparison of goodness-of-fit of linear and Weibull models for the survival curves of *E. coli, L. innocua* and *S. aureus* in black tiger shrimp.

**Bacterial species**	**Temperature (°C)**	**Pressure (MPa)**	**The log-linear model**			**The fitted Weibull model**			**The simplified Weibull model**		
			**RMSE**	***A_*f*_***	***B_*f*_***	**RMSE**	***A_*f*_***	***B_*f*_***	**RMSE**	***A_*f*_***	***B_*f*_***
*E. coli*	30	300	0.211	1.259	1.159	0.004	1.023	1.017	0.001	1.007	1.003
		400	0.062			0.007			0.002		
		500	0.081			0.009			0.002		
		600	0.141			0.008			0.005		
	40	300	0.231	1.233	1.168	0.010	1.021	0.998	0.008	1.013	1.005
		400	0.050			0.003			0.004		
		500	0.192			0.010			0.007		
		600	0.133			0.010			0.005		
*L. innocua*	30	300	0.092	1.259	1.209	0.002	1.011	0.979	0.002	1.008	0.999
		400	0.024			0.004			0.002		
		500	0.015			0.004			0.005		
		600	0.022			0.002			0.003		
	40	300	0.026	1.232	1.193	0.005	1.007	0.987	0.004	1.002	0.997
		400	0.145			0.030			0.017		
		500	0.056			0.007			0.008		
		600	0.043			0.003			0.003		
*S. aureus*	30	300	0.016	1.190	1.065	0.003	1.008	0.986	0.007	1.009	1.002
		400	0.073			0.004			0.006		
		500	0.122			0.001			0.001		
		600	0.091			0.002			0.004		
	40	300	0.073	1.174	1.146	0.001	1.002	0.980	0.002	0.998	0.991
		400	0.044			0.003			0.004		
		500	0.056			0.007			0.005		
		600	0.053			0.003			0.002		
	50	300	0.022	1.182	1.157	0.005	1.008	0.985	0.007	1.003	0.999
		400	0.097			0.030			0.011		
		500	0.110			0.007			0.008		

Generally, higher *R*^2^ values, smaller RMSE values, and *A*_*f*_ and *B*_*f*_ values that are closer to 1 indicate a better model fit. When β′ was fixed at 1 for the entire *P–T* domain, the adjusted *R*^2^ for all the microbial groups varied between 0.76 and 0.91. In contrast, the fitted Weibull curves each had an adjusted *R*^2^ ≥ 0.94 and very small RMSE values (the maximum, 0.03, was obtained for both *L. innocua* and *S. aureus*). However, the simplified Weibull models had adjusted *R*^2^ and RMSE values ranging from 0.97 to 0.99 and 0.001 to 0.017, respectively.

**Figure 3:**
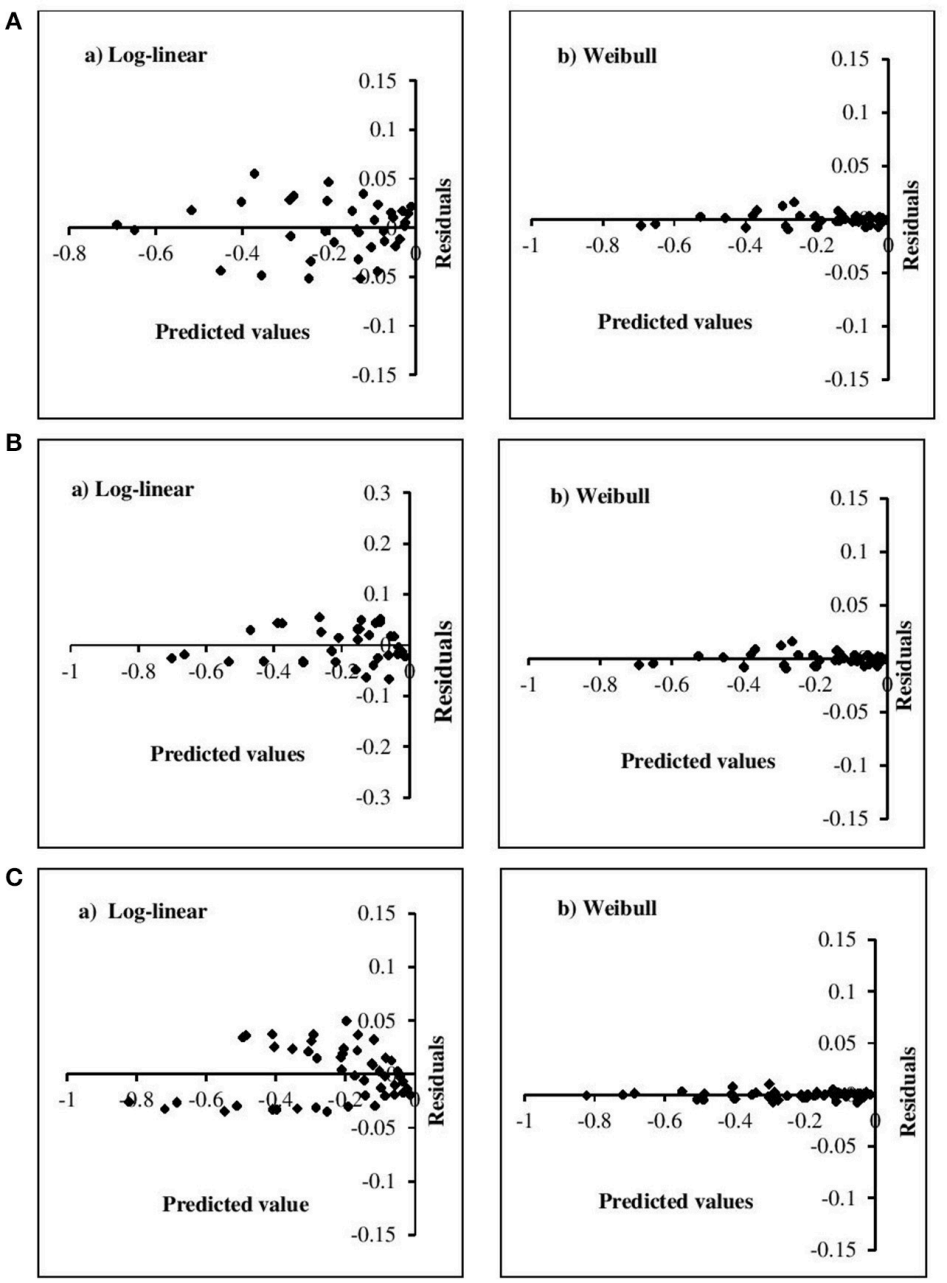
Residual plots for the inactivation of **(A)** *E. coli*, **(B)** *L. innocua*, **(C)** *S. aureus* for log-linear and simplified Weibull model.

**Figure 4:**
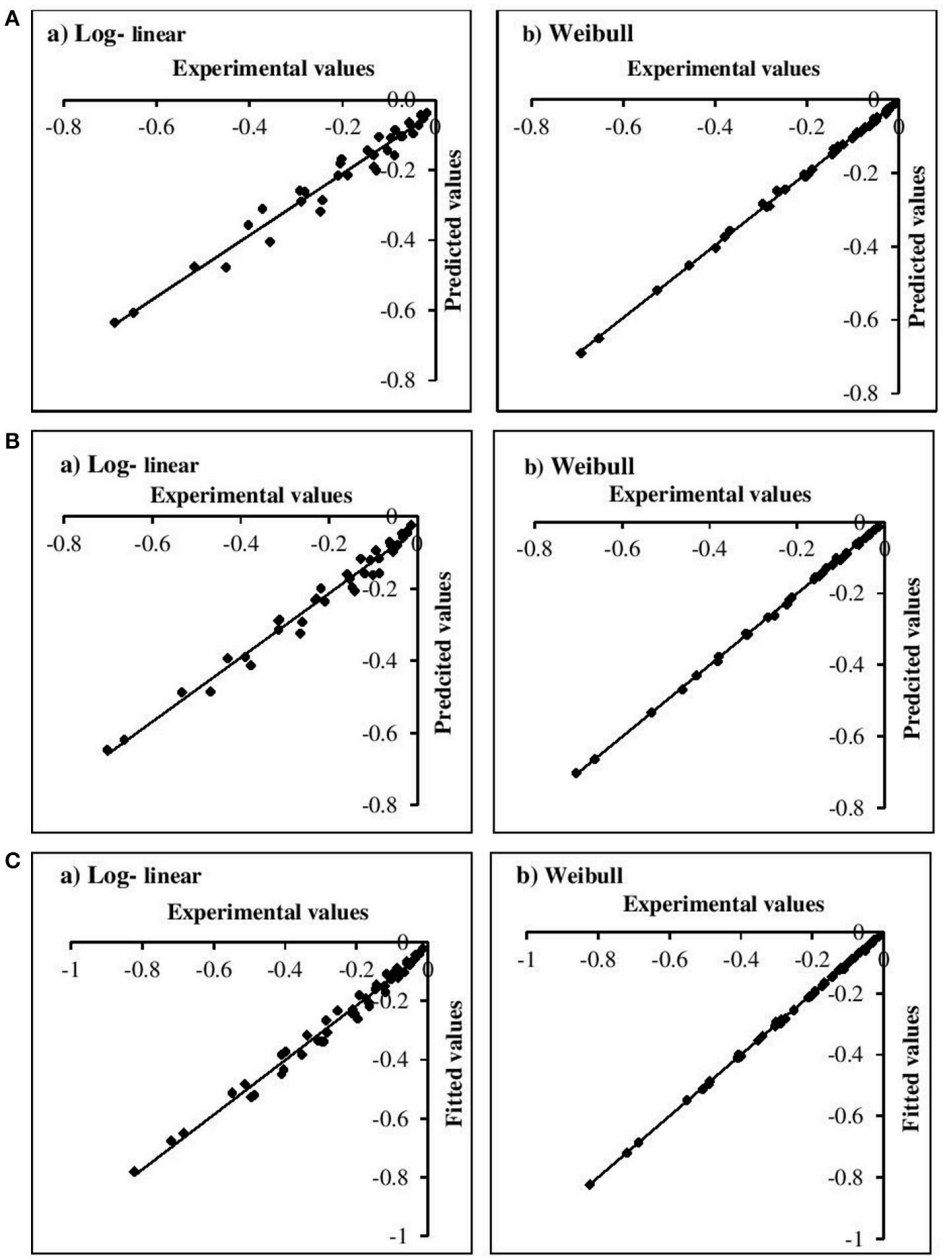
Correlation between experimental and predicted values for **(A)** *E. coli*, **(B)** *L. innocua*, **(C)** *S. aureus* for log-linear and simplified Weibull model.

The adequacy of the model fitting was investigated using residual plots (Figure 3) and calculating the correlation between the predicted and experimental values for the linear and non-linear models at the fixed β values (Figure 4). Residual plots indicate whether a model is fully appropriate for the data being analyzed. The residual plots strongly suggested that the linear regression function was not appropriate, as there was curvature in the data and the residuals departed from 0 in a systematic fashion. However, for the non-linear models, the residuals were distributed randomly, falling within a horizontal band centered around 0. Moreover, the correlation between the predicted and experimental values also indicated a close relationship between these values for the non-linear models, which proved to be more appropriate than the log-linear models. In addition, the Weibull model has previously been successfully used to describe microbial kinetics for various HPP products (Van Opstal et al., 2003; Pilavtepe-Çelik et al., 2009; Serment-Moreno et al., 2016), with the inactivation curves demonstrating upward or downward concavity.

### Effect of HPP on the scale parameter (δ) and rate constant (k)

The deviation of the refitted scale parameters (δ values) from the corresponding δ′ values was also investigated (Table 4). At the fixed β values, the δ values for all three pathogens were almost comparable to the δ′ values. However, at β = 1, the difference between the δ_*l*_ and δ values was significant (*P* < 0.05). A non-linear relationship between the δ values and the applied pressure and temperature was observed. Therefore, using first-order kinetics would lead to an underestimation or overestimation of the decimal reduction time.

**Table 4:** Scale factors (δ′, δ, and δ_*l*_) obtained after model fitting at different pressure-temperature combinations for different pathogens in black tiger shrimp.

**Bacterial species**	**Pressure (MPa)**	**δ values at different temperatures (min)**								
		**30°C**			**40°C**			**50°C**		
		**δ′**	**δ**	**δ_*l*_**	**δ′**	**δ**	**δ_*l*_**	**δ′**	**δ**	**δ_*l*_**
*E. coli*	300	57.46 ± 3.24^aA^	55.15 ± 2.60^aA^	141.94 ± 9.74^bA^	32.08 ± 0.94^cA^	35.29 ± 1.64^cA^	57.58 ± 3.24^aA^	ND	ND	ND
	400	45.92 ± 2.07^aB^	46.29 ± 1.14^aB^	83.38 ± 6.31^bB^	23.28 ± 1.31^cB^	23.58 ± 0.88^cB^	31.57 ± 1.33^dB^	ND	ND	ND
	500	34.19 ± 2.24^aC^	35.25 ± 2.21^aC^	58.33 ± 4.77^bC^	15.35 ± 0.90^cC^	15.54 ± 1.00^cC^	18.90 ± 1.54^dC^	ND	ND	ND
	600	27.50 ± 1.10^aD^	28.12 ± 1.19^aD^	42.10 ± 2.78^bD^	12.10 ± 0.84^cD^	12.35 ± 0.91^cD^	14.85 ± 1.25^cD^	ND	ND	ND
*L. innocua*	300	53.85 ± 3.21^aA^	52.15 ± 4.40^aA^	128.41 ± 7.14^bA^	34.97 ± 1.22^cA^	34.31 ± 1.89^cA^	52.07 ± 3.04^aA^	ND	ND	ND
	400	44.75 ± 3.07^aB^	43.48 ± 3.22^aB^	75.15 ± 3.24^bB^	23.18 ± 1.14^cB^	23.25 ± 2.04^cB^	30.81 ± 2.71^dB^	ND	ND	ND
	500	33.79 ± 2.00^aC^	34.47 ± 2.34^aC^	52.64 ± 2.48^bC^	15.50 ± 1.07^cC^	15.11 ± 1.11^cC^	18.55 ± 0.94^cC^	ND	ND	ND
	600	27.46 ± 1.13^aD^	27.43 ± 1.16^aD^	38.20 ± 1.04^bD^	12.10 ± 0.83^cD^	12.09 ± 1.00^cD^	14.54 ± 0.76^cD^	ND	ND	ND
*S. aureus*	300	61.46 ± 4.59^aA^	63.72 ± 4.66^aA^	139.27 ± 8.14^bA^	36.72 ± 2.22^cA^	37.31 ± 1.24^cA^	47.50 ± 2.11^dA^	21.45 ± 3.04^eA^	20.08 ± 2.24^eA^	29.05 ± 3.22^fA^
	400	54.06 ± 0.04^aB^	53.26 ± 2.26^aB^	83.41 ± 5.02^bB^	27.60 ± 1.06^cB^	29.83 ± 1.55^cB^	36.34 ± 2.94^dB^	14.81 ± 2.54^eB^	14.48 ± 1.32^eB^	17.33 ± 1.10^eB^
	500	38.76 ± 0.04^aC^	39.43 ± 2.43^aC^	56.47 ± 4.66^bC^	17.47 ± 1.64^cC^	17.86 ± 1.79^cC^	19.76 ± 1.13^cC^	7.82 ± 1.84^dCA^	7.30 ± 0.89^dC^	10.39 ± 1.07^eC^
	600	29.48 ± 0.04^aD^	30.44 ± 3.06^aD^	40.37 ± 3.13^bD^	14.01 ± 1.44^cD^	15.54 ± 1.94^cD^	16.88 ± 2.84^cD^	ND	ND	ND

*All values are means ± standard deviations of data from two independent experiments. Different superscripts (a, b, c, d, e, f) in the same row indicate significant difference (P < 0.05). Different superscripts (A, B, C, D) in the same column indicate significant difference (P < 0.05)*.

An increase in pressure resulted in a reduction in δ for all three pathogens. Similarly, at constant pressure, an increase in temperature led to a decrease in δ. The reduction in δ values with increasing pressure and temperature revealed that both parameters contributed additively or synergistically to the death of the bacteria. Increased pressure combined with increased temperature targets many factors in microbial cells rather a single factor, such as, membranes, ribosomes, nucleic acids, proteins (such as, enzymes), and so on. Hence, it is hard to separate the individual lethality of each processing parameter (Smelt et al., 2001).

The death rate constant (*k*, min^−1^) for the three pathogens was dependent on both pressure and temperature. The pressure sensitivity of *E. coli, L. innocua*, and *S. aureus* were estimated using the activation volume, *V*_*a*_. The *V*_*a*_ in microbial death kinetics represents the formation rates of an activated complex or the quasi-state equilibrium that is generally favored by compaction (Chakraborty et al., 2015). The decrease in *V*_a_ with increased temperature suggested an additive or synergistic effect of pressure and temperature on the microbial death rate. At all temperatures, the *V*_*a*_ values were negative, ranging between −5.65 and −8.11 cm^3^ mol^−1^ for *E. coli*, −6.30 and −9.11 cm^3^ mol^−1^ for *L. innocua*, and −5.04 and −11.43 cm^3^ mol^−1^ for *S. aureus* (Table 5). This indicates that pressure has a lethal effect on the pathogens. In general, a large negative *V*_a_ value signifies a higher responsiveness to changes in pressure (Mussa et al., 1999). In the current study, the *V*_a_ values became more negative with increase in temperature, indicating that the pathogens became more responsive to pressure changes at increasing temperatures.

**Table 5:** Inactivation rate constants, activation volume, and activation energy values for *E. coli, L. innocua* and *S. aureus* using Eq. (5–7).

**Bacterial species**	**Pressure (MPa)**	***k*-value (×10^−2^ min^−1^)**			***E_*a*_* (kJ mol^−1^)**
		**30°C**	**40°C**	**50°C**	
*E. coli*	300	3.19 ± 0.11^aA^	4.98 ± 0.27^bA^	ND	44.63 ± 2.11^A^
	400	3.80 ± 0.47^aAB^	7.46 ± 0.69^bAB^	ND	47.87 ± 3.24^A^
	500	4.99 ± 0.31^aBC^	11.31 ± 1.37^bBC^	ND	61.75 ± 4.46^B^
	600	6.31 ± 0.66^aC^	14.23 ± 1.89^bC^	ND	64.71 ± 5.04^B^
−*V_*a*_* (cm^3^ mol^−1^)		5.65 ± 0.04^a^	8.11 ± 0.04^b^	ND	
*L. innocua*	300	3.48 ± 0.68^aAB^	5.29 ± 0.41^bA^	ND	46.92 ± 3.21^aA^
	400	4.17 ± 0.39^aAB^	7.81 ± 0.79^bAB^	ND	50.46 ± 6.84^aA^
	500	5.26 ± 0.56^aBC^	12.01 ± 0.83^bBC^	ND	63.26 ± 4.96^aB^
	600	6.62 ± 0.62^bC^	15.01 ± 1.75^bC^	ND	65.89 ± 3.13^aB^
−*V_*a*_* (cm^3^ mol^−1^)		6.30 ± 0.24^a^	9.11 ± 0.51^b^	ND	
*S. aureus*	300	2.98 ± 0.16^aA^	5.09 ± 0.59^bA^	9.46 ± 1.13^aA^	39.77 ± 1.93^aC^
	400	3.57 ± 0.39^aAB^	6.37 ± 0.48^bA^	13.12 ± 1.42^aA^	41.95 ± 2.76^aAC^
	500	4.82 ± 0.87^aBC^	10.64 ± 0.93^bBC^	26.02 ± 2.18^aB^	52.90 ± 4.45^aD^
	600	6.15 ± 0.82^aC^	12.23 ± 1.01^bBC^	ND	63.12 ± 6.59^aB^
−*V_*a*_* (cm^3^ mol^−1^)		5.04 ± 0.87^a^	7.81 ± 1.04^b^	11.43 ± 2.16^c^	

*All values are means ± standard deviations of data from two independent experiments. Different superscripts (a, b, c) in the same row indicate significant difference (P < 0.05). Different superscripts (A, B, C, D) in the same column indicate significant difference (P < 0.05). ND not determined*.

The temperature sensitivity of all three pathogens was estimated by computing the activation energy, *E*_*a*_, at fixed pressures. The *E*_*a*_ values increased from 44.63 to 64.71 kJ mol^−1^, 46.92 to 65.89 kJ mol^−1^, and 39.77 to 63.12 kJ mol^−1^ for *E. coli, L. innocua*, and *S. aureus*, respectively, as the pressure was increased from 300 to 600 MPa (Table 5). This signified that the temperature sensitivity of the pathogens is higher at higher pressures. At lower pressures (300–400 MPa), the *E*_*a*_ values for the three pathogens were not significantly different (*P* ≥ 0.05). Similarly, no significant difference in the *E*_*a*_ values (*P* ≥ *0.05*) was observed for pressure treatments of 500 and 600 MPa. The temperature sensitivity of the inactivation rate within a particular temperature domain varies considerably between species and even between bacterial strains. Previous studies have revealed that the temperature sensitivity (according to *E*_*a*_ values) of the inactivation rate of various microorganisms varied greatly at different pressures (Alpas et al., 1999; vanBoekel, 2002). The higher *V*_a_ and lower *E*_*a*_ values obtained in the present study might be due to the separation of the SPPE from the pressure-hold effect, which was not investigated in the earlier studies.

## Conclusions

In this study, the effects of high pressure and temperature on the inactivation kinetics of *E. coli, L. innocua*, and *S. aureus* in black tiger shrimp were investigated. An additive or synergistic effect of pressure and temperature on the inactivation of the pathogens was noticed in the ranges of processing parameters studied. *S. aureus* was found to be the most baro-resistant species among the three pathogens. The minimum processing intensity required for complete destruction of *S. aureus* was 500 MPa/9 min/50°C. The study showed that the sensitivity of the microorganisms to the applied conditions was different during single-pulse and pressure-hold pressurization. The methodology used in this study could be used to develop a simplified Weibull models to describe and predict non-linear survival curves of bacteria in other foods and of other microorganisms. Accurate prediction of survival curves at different pressures and temperatures would be beneficial to the food industry in terms of optimum selection of processing conditions to achieve the desired levels of bacterial inactivation, while also minimizing the production costs and maintaining a high degree of nutritional quality and good flavor and texture.

## Chemicals used in this study

Propylene glycol (PubChem CID: 1030).

Peptone (bacteriological) (PubChem CID: 9257).

## Author contributions

All authors listed have made a substantial, direct and intellectual contribution to the work, and approved it for publication.

### Conflict of interest statement

The authors declare that the research was conducted in the absence of any commercial or financial relationships that could be construed as a potential conflict of interest.

## Abbreviations

HPP: High-pressure processing
*E*_*a*_: Activation energy
MPa: Megapascal
*N*_*0*_: Initial microbial counts in the control samples
*N*_*PE*_: Number of cells that survived after single-pulse pressurization
*N*_*t*_: Number of cells that survived after pressure treatment for time *t* (min)
SPPE: Single-pulse pressure effect
*V_a_*: Activation volume.
